# Protic ionic liquids with primary alkylamine-derived cations: the dominance of hydrogen bonding on observed physicochemical properties†

**DOI:** 10.1039/c8ra00402a

**Published:** 2018-03-09

**Authors:** Mahfuzul Hoque, Morgan L. Thomas, Muhammed Shah Miran, Mio Akiyama, Mayeesha Marium, Kazuhide Ueno, Kaoru Dokko, Masayoshi Watanabe

**Affiliations:** Department of Chemistry and Biotechnology, Yokohama National University 79-5 Tokiwadai, Hodogaya-ward Yokohama 240-8501 Japan mwatanab@ynu.ac.jp

## Abstract

Novel protic ionic liquids (PILs) were synthesized by neutralization of primary alkylamines with bis(trifluoromethanesulfonyl)amide acid. An extensive hydrogen bonding network in these PILs was observed *via* lower thermal stability, temperature dependent inversion from non-Newtonian to Newtonian fluidic behavior, and lower ionicity compared to their secondary and tertiary analogues.

## Abstract 2

The dominance of hydrogen bonds (networking) over the physicochemical features of primary alkylamine-PILs based on an amide acid.

## Graphical abstract

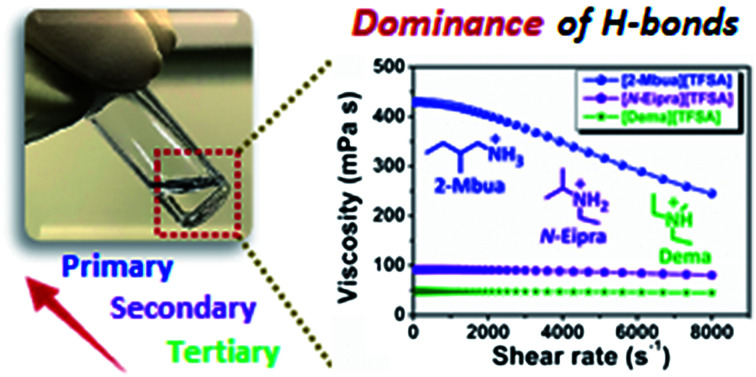

## Introduction

Ionic liquids (ILs) are defined as liquids composed solely of ions (at the temperature of interest),^1^ and research has evolved to incorporate several classes such as aprotic ionic liquids (AILs), protic ionic liquids (PILs), solvate ionic liquids (SILs) and related categories such as solvent-in-salt systems and mixtures *etc.*^1,2^ Every variant of ILs has merits and shortcomings; their usefulness/functionality is dictated by their tunable fundamental properties.^2,3^ In this robust branch of liquid chemistry, PILs are also unique in that they are easily synthesized *via* a stoichiometric Brønsted acid–base reaction involving proton transfer, in contrast to AILs.^2^ The PIL ethylammonium nitrate (EAN),^4^ is considered one of the earliest reported room-temperature ILs. Extensive research has been performed to understand its fundamental properties.^5–8^ Ludwig *et. al.*^9^ demonstrated that EAN forms a dense, co-operative hydrogen (H–) bonding network akin to water. Further study of H-bonding and its effects on the features of PILs beyond the EAN system is attractive. Despite its intriguing properties, EAN suffers from poor thermal stability^9^ owing to the relatively low Δp*K*_a_^10,11^ and electrochemical instability in EAN–water binary mixtures due to the unstable nature of the anion.^12^ However, beyond EAN and related combinations, further research has been hindered by the scarcity of room temperature liquid samples (or even low melting temperature salts).^3^ Incorporation of more weakly Lewis-basic anions has opened new opportunities for alkylamine-based PILs, in particular for tertiary amines, and also for secondary amines.^2,3^ Owing to the importance of hydrogen bonding networks in PILs (*vide supra*), further study of primary alkylamine-based PILs, with a higher number of potential H-bond donors, is also attractive. Moreover, primary alkylamine-PILs are promising for application in various areas, including, but not limited to: as non-aqueous solvents in molecular self-assembly of macromolecules,^3^ as reagents/media in protein chemistry,^13^ as precursors of nitrogen doped carbon materials,^14^ in tribological applications^15^ and in electrochemical devices.^2^ Thus, from both a fundamental and applied perspective, primary alkylamine-based PILs with high Δp*K*_a_ are an appealing system. However, they are rarely reported in comparison with tertiary alkylamine-PILs owing to the synergy of intermolecular forces,^5,6^ such as doubly ionic H-bonds,^9,16^ and efficient packing of the ions promoting crystallization at relatively high temperatures comparable to that of molecular liquids such as water.^5,9^

In this article, we report several selected prototype examples of novel primary amine-PILs using branched alkylammonium cations and bis(trifluoromethanesulfonyl)amide ([TFSA]^−^) as anion. Herein, we present the structure–property relationships for the variation in cationic structure of 2-ethylhexylammonium ([2-Ehexa]^+^), 2-methylbutylammonium ([2-Mbua]^+^), and 2-methylpropylammonium ([2-Mpra]^+^). Furthermore, secondary and tertiary isomers of [2-Mbua]^+^, namely *N*-ethylisopropylammonium ([*N*-Eipra]^+^), and diethylmethylammonium ([Dema]^+^) were compared. Structures of cations and anions are shown in Scheme S1 (ESI†).

## Results and discussion

First, thermal analyses in combination with FT-IR and NMR spectroscopy were performed to probe the N–H bond strength to provide detail on the effect of networking H-bonds.

**Fig. 1:**
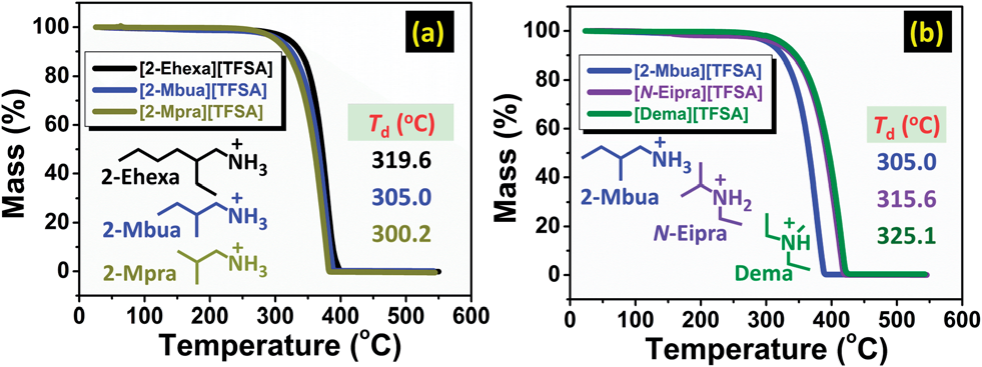
Thermo-gravimetric curves for PILs with the variation in (a) alkyl chain length, and (b) number of hydrogen bond donors.

The thermal decomposition temperature (*T*_d_, see ESI†) was higher for [2-Ehexa][TFSA] than the PILs with shorter alkyl chain in their cationic structure as shown in Fig. 1a.

Moreover, it was lower for [2-Mbua][TFSA] (Fig. 1b) than its isomeric counterparts. In general, the thermal stability of such PILs are superior to the EAN series due to high Δp*K*_a_,^10,11^ but inferior to tertiary amine-PILs with alkylammonium cationic structure.^17^ The change in *T*_d_ for primary alkylamine-PILs with different cations was relatively small owing to the somewhat similar Δp*K*_a_. We further investigated [2-Ehexa]^+^ with other anions providing greater variability in Δp*K*_a_ (see Fig. S1 and Table S1, ESI†), which was congruent with the previous report in which *T*_d_ was greatly influenced by Δp*K*_a_ because of the change in the strength of N–H bonds.^18^ Thus, regarding TG for variation of cationic structure, we could infer that small variations in thermal stability are related to the perturbation in the weaker interactions, for example H-bonds, rather than stronger interactions *i.e.* N–H bond.

**Fig. 2:**
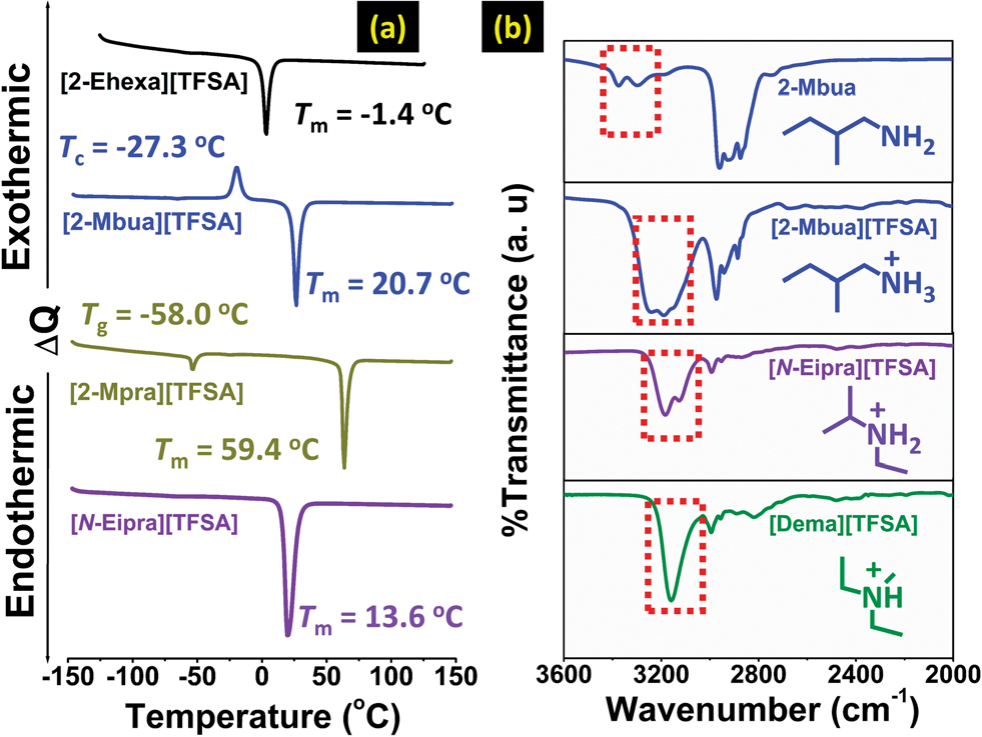
(a) DSC patterns of PILs, (b) FT-IR spectra of the neutral amine 2-Mbua and PILs.

Furthermore, DSC measurements revealed an interesting change in phase behaviour for the primary alkylamine-PILs as shown in Fig. 2a, S2 and S3 (ESI†). Reduction in alkyl chain length increased the melting point (Fig. 2a), attributed to the decrease of asymmetry of cation contributing to efficient ion-packing.^11^ Also, cations with smaller and less symmetrical alkyl chains (here [2-Mpra][TFSA]) increase the enthalpy of fusion (Δ*H*_f_) due to the enhanced cation–anion ion pair interaction energy (per mole) of PILs as previously observed for AILs.^11^ Here we again note that we further considered the role of the anion for [2-Ehexa]^+^ (see Fig. S2 and S3 and related discussion, ESI†). Also, reduction in the number of H-bond donors (primary amine to secondary amine) resulted in melting point suppression (Fig. 2a and S4, ESI†) as the possibility of forming networking H-bonds^9,16^ was lessened. However, considering previous reports in AILs,^19^ H-bonding promoted fluidity and reduction in melting points. So, primary amine-PILs might represent a borderline system between PILs and molecular liquids, whereas tertiary amine-PILs could be considered more like AILs. This shift is presumably driven by subtle changes in the relative contributions of intermolecular interactions such as H-bonding, London forces *etc.* in these coulombic fluids as reported in earlier research.^20^ Overall, these DSC results indicate that networking H-bonds might have a greater effect in primary amine-PILs.

FT-IR spectra of the PILs are shown in Fig. 2a. [2-Mbua][TFSA] exhibited a broad band corresponding to symmetric and asymmetric N–H stretching.^21^ The broad band was comprised of maxima cantered at ∼3200 cm^−1^ flanked by two shoulder peaks around ∼3260 cm^−1^, and ∼3140 cm^−1^ respectively. A similar spectral pattern was observed for EAN.^8^ To confirm the assignment of the bands of [2-Mbua][TFSA], the FT-IR spectrum of the (neutral) primary amine, 2-Mbua was also recorded exhibiting only two broad bands at ∼3380 cm^−1^ and ∼3290 cm^−1^.^21^ Thus, the additional band in [2-Mbua][TFSA] could arise due to the N–H bonded species introduced *via* protonation. On the other hand, the secondary alkylamine-PIL, [*N*-Eipra][TFSA] exhibited two distinct peaks at ∼3190 cm^−1^ and ∼3130 cm^−1^.^21^ And, the tertiary alkylamine-PIL, [Dema][TFSA] showed only a narrow single peak at 3159 cm^−1^ for the N–H bond.^22^ For the longer chain cation in [2-Ehexa][TFSA], similar findings were obtained (Fig. S5, ESI†). If we consider the most intense N–H peak (Fig. 2a and S5, ESI†), then in changing from a tertiary to primary amine cation, the N–H peak position shifted a little to higher wavenumber.^23^ However, exact assignment of these bands to the specific chemical species is challenging due to the existence of rotational isomers of primary amine.^23^^1^H-NMR spectra may support multiple H-bonds observed in FT-IR spectra for primary alkylamine-PILs, exhibiting the single N–H proton peak^24^ (Fig. S6, ESI†) that indicates the time scale of NMR is sufficiently long to average the multiple H-bonds. Note that the chemical shift of [2-Mbua][TFSA] is 5.84 ppm, which is lower than [*N*-Eipra][TFSA] (5.93 ppm) and [Dema][TFSA] (6.72 ppm). The multiple H-bonds in [2-Mbua][TFSA] are averaged, and thus, the averaged electron density of N–H protons could become higher for [2-Mbua][TFSA]. This is also evident from the FT-IR spectra, where the lower wavenumber limit of the broad peaks in [2-Mbua][TFSA] and [*N*-Eipra][TFSA] do not appear at higher wavenumber than that of [Dema][TFSA], despite the previously discussed trend (*vide supra*) in the peak maxima. Similar proton chemical shifts were observed for all the primary alkylamine-PILs *i.e.* [2-Ehexa][TFSA], [2-Mbua][TFSA], and [2-Mpra][TFSA]. Generally, a large downfield shift for the N–H proton was demonstrated with the change in anion basicity/Δp*K*_a_.^24^ But, here, the difference in Δp*K*_a_ was marginal.

Finally, rheological study coupled with transport properties as a function of temperature were performed to gain in-depth understanding about the dominant effect of H-bonds. Then, Walden plot analysis was performed to reveal the effect of such inter-ionic interaction over the ionic nature of primary alkylamine-PILs.

**Fig. 3:**
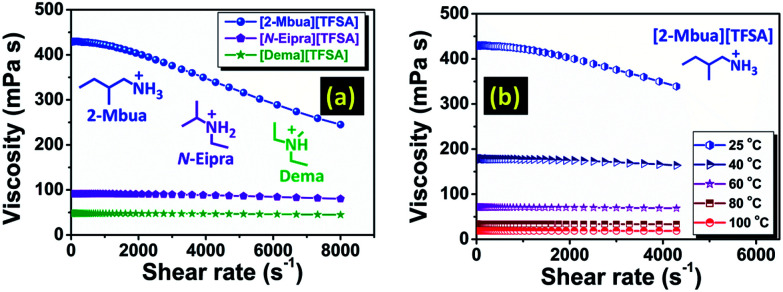
Change of viscosity as a function of shear rate (a) at 25 °C, (b) under various temperatures. For (b), shear rate was limited to 4000 s^−1^ at higher temperatures due to the expelling of the samples at very high shear rate.

Stress controlled rheometric analysis has been perceived to be useful to unravel the role of H-bonding in ILs.^25^ Herein, at zero shear rate, viscosity of [2-Mbua][TFSA] was approximately four and eight times higher than [*N*-Eipra][TFSA] and [Dema][TFSA] respectively at 25 °C (Fig. 3a). Interestingly, [2-Mbua][TFSA] exhibited a rapid decrease of viscosity above a shear rate of ∼570 s^−1^; an indication of shear thinning phenomena.^26^ The onset of shear thinning for [TFSA]^−^ based primary amine-PIL was higher than EAN, EAF (ethylammonium formate), and PAN (propylammonium nitrate) (above ∼100 s^−1^).^26^

The onset of shear thinning has been reported to be dependent on the number of H-bonds in PILs.^27^ The usage of [TFSA]^−^ ((CF_3_SO_2_)_2_N^−^) as anion could provide multiple sites for H-bonding which includes both O atoms^28^ in the –SO_2_– moieties as well as the N^−^ (ref. 28) in contrast with NO_3_^−^ in EAN.^5,9^ So, an increment in the onset of shear thinning is indicative of the build-up of denser H-bonding networks in the current series of PILs compared to EAN. Additionally, the onset of shear thinning for [2-Ehexa][TFSA] (∼950 s^−1^) was increased as shown in Fig. S7 (ESI†). For the EAN series, the onset value of shear thinning was correlated to their nanostructure in which the anion had less effect and cations with longer chains generated better defined nanostructure (bicontinuous microemulsions or more closely corresponding to L_3_ (sponge) phases) without affecting its type.^29^ The nanostructure of the current systems is still unknown. But, it could be assumed that the concomitant increases in the onset of shear thinning for current [TFSA]^−^ based primary alkylamine-PILs could be associated with the amphiphilicity of the cation leading to the increased degree of segregation between polar and apolar domains and thus a well-defined nanostructure.^29^ On the contrary, [*N*-Eipra][TFSA] displayed only a meagre loss of viscosity with shear rate, and for [Dema][TFSA] it was almost invariant akin to the observation of Separovic *et al.*^30^ for tertiary amine-PILs. Overall, this is the first conspicuous observation of the effect of networking H-bonds in primary amine-PILs with high Δp*K*_a_, as previously reported for functionalized PILs containing free/pendant –OH, –NH_2_ and –SH groups.^31^ As we know, although being coulombic fluids, in PILs, there is a competition between the non-covalent interactions such as H-bonding, London forces *etc.*^29^ As evidenced from the rheological analysis, dominancy of H-bonding could drastically shift the relative contribution of these forces. Therefore, at low temperature, primary amine-PILs exhibit properties resembling molecular liquids, but at high temperature resembling those of AILs.

**Fig. 4:**
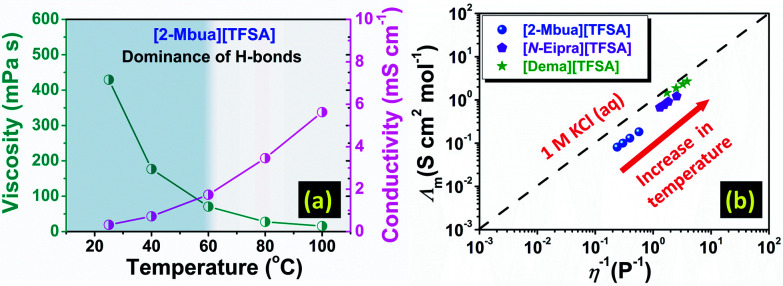
(a) Change of viscosity and conductivity as a function of temperature, (b) Walden plot (25–40 °C).

The presence of H-bonds in ionic liquids is quite compelling and especially for PILs.^16^ Like H-bonds in conventional media, they are quite responsive to external stimuli, such as temperature.^30^ Under temperature variation, [2-Mbua][TFSA] displayed a change in shear thinning from ‘strong’ to ‘no thinning’ as shown in Fig. 3b. It was noticeable that from 60 °C, shear thinning was absent supporting the intrinsic non-Newtonian character of primary amine-PILs like EAN (Fig. 3b). Thus, microscopically, the local structure of [2-Mbua][TFSA] was strongly influenced by the short-range networking H-bonds controlling its macroscopic characteristics. During temperature increase, however, those short-range interactions become weaker owing to the thermal perturbations finally resulting in a liquid structure dominated by the coulombic interactions and London forces instead of networking H-bonds. Such temperature-tuneable transitions of long range forces in PILs have also been highlighted recently by Rutland *et al.*^7^ for EAN. This further demonstrates the unique but more perplexing micro-heterogeneity in PILs, especially for primary amine-PILs which require further investigation. As demonstrated in Fig. 4a, for [2-Mbua][TFSA], viscosity decreased more rapidly from 25 °C to 60 °C than from 60 °C to 120 °C. Conversely, the change in conductivity showed the opposite trend (Fig. 4a). Similar behaviour was noticed for [*N*-Eipra][TFSA] (Fig. S8 and S9, ESI†) but at below and above 40 °C in accordance with the rheological responses. So, the PILs can switch their non-Newtonian fluidic character at a certain threshold temperature pertaining to their degree of H-bonding. The order of maximum temperature for shear thinning was primary (60 °C) > secondary (40 °C) > tertiary (not applicable). Therefore, the order of number of networking H-bonds could be primary > secondary > tertiary (Fig. S4, ESI†).

Considering the above discussion, such a high extent of inter-ionic interactions could possibly affect the ionicity of primary amine-PILs, which was further explored *via* Walden plot analysis.^32^ As shown in Fig. 4b, [2-Mbua][TFSA] lied farthest from the ideal KCl line. In comparison, [*N*-Eipra][TFSA] was much closer to the ideal line (Fig. 4b) than [2-Mbua][TFSA]. And, [Dema][TFSA] was located just beneath the ideal line as per the previous study.^33^ The extent of the deviation from the Walden plot could be a rough estimation of ionicity typically known as the Walden rule.^34^ Ideally, PILs should be closer to the ideal KCl line, because, at high Δp*K*_a_, proton transfer is almost complete, and more so in primary amine-PILs even at low Δp*K*_a_ (10).^35^ Instead, in [TFSA]^−^ based primary amine-PILs ionicity was lowered, we speculate that this may be due to the distinct segregation of ions into polar and apolar domains stemming from enhanced networking H-bonds. A decrease in ionicity with increasing nanophase separation has been observed in typical imidazolium-based AILs.^36^ Such an effect could be diminished in reducing the number of H-bond donors from primary amine-PILs to tertiary amine-PILs, and in turn enhancing the ionicity of PIL system (Fig. 4b). [2-Ehexa][TFSA] also exhibited subionic behaviour displaying lower ionicity in resemblance to [2-Mbua][TFSA] as shown in Fig. S10.† Such a low degree of ionicity for primary amine-PILs with longer alkyl chains was also noted for nitrate anion ([NO_3_]^−^) based PILs such as BAN (butylammonium nitrate).^37^ Observation of shear thinning also supported the deviation in the Walden plot for primary amine-PILs (Fig. 3a and S7†). Thus, the results of this work further invoke the common perception about the ionic nature of PILs, which was also highlighted in a recent study.^7^

## Conclusions

In closing, a novel series of primary alkylamine-PILs were successfully synthesized *via* judicious selection of amines with [TFSA]^−^ anion. *Via* systematic variation in the cationic structures, we conclude that networking H-bonds unambiguously dictated the observed physicochemical properties of such PILs, for instance lower thermal stability, temperature driven inversion of non-Newtonian behaviour, and lower ionicity. These findings are quite fascinating and will surely ignite new interest in ionic liquid community to further explore the properties and microstructures of this important class of PILs, reminiscent of EAN, for task-specific applications.^38^

## Conflicts of interest

There are no conflicts to declare.

## Supplementary Material

RA-008-C8RA00402A-s001
