# Supplementary material for: Protic ionic liquids with primary alkylamine-derived cations: the dominance of hydrogen bonding on observed physicochemical properties
Source: RSC Adv. 2018 Mar 9;8(18):9790–4. doi: 10.1039/c8ra00402a (PMC9078737; doi:10.1039/c8ra00402a)
Supplement: RA-008-C8RA00402A-s001 [file RA-008-C8RA00402A-s001.pdf]

Electronic Supplementary Material (ESI) for RSC Advances.
This journal is © The Royal Society of Chemistry 2018

Electronic Supplementary Information:

Protic ionic liquids with primary alkylamine-derived cations: dominance of hydrogen bonding on observed physicochemical properties

Mahfuzul Hoque, Morgan L. Thomas, Muhammed Shah Miran,‡ Mio Akiyama, Mayeesha Marium, Kazuhide Ueno, Kaoru Dokko, and Masayoshi Watanabe*

[a]Department of Chemistry and Biotechnology, Yokohama National University, 79-5 Tokiwadai, Hodogaya-ward, Yokohama 240-8501, Japan.

‡Permanent address: Department of Chemistry, University of Dhaka, Dhaka 1000, Bangladesh.

**E-mail: mwatanab@ynu.ac.jp*

Primary, secondary, and tertiary amines/Cations

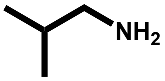

2-Methylpropylamine
2-Methylpropylammonium
[2-Mpra]$^+$

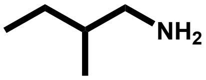

2-Methylbutylamine
2-Methylbutylammonium
[2-Mbua]$^+$

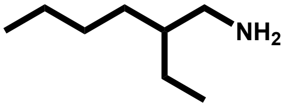

2-Ethylhexylamine
2-Ethylhexylammonium
[2-Ehexa]$^+$

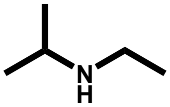

N-Ethylisopropylamine
N-Ethylisopropylammonium
[N-E*i*pra]$^+$

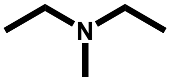

Diethylmethylamine
Diethylmethyammonium
[Dema]$^+$

Acids/Anions

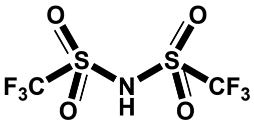

Bis(trifluoromethanesulfonyl) amide acid
Bis(trifluoromethanesulfonyl) amide
[TFSA]$^-$

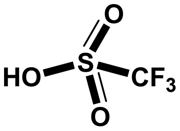

Trifluoromethanesulfonic acid
Trifluoromethanesulfonate
[TFO]$^-$

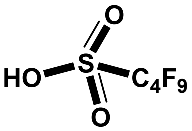

Nonafluorobutanesulfonic acid
Nonafluorobutanesulfonate
[NFO]$^-$

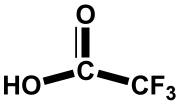

Trifluoroacetic acid
Trifluoroacetate
[TFA]$^-$

Scheme S1. Structure of amines and acids employed in this study.

Experimental details

Synthesis of protic ionic liquids/protic salts

All the amines (TCI, Japan) and acids (Wako Chemicals, Japan), also shown in Scheme. S1, were used as received. Prior to the synthesis, reagents were weighed and handled inside a dry box (Daikin, Japan). Glassware was dried in an oven at 80 ^oC before carrying out the synthesis under an inert atmosphere.

The PILs were synthesized by mixing amine and acid in a 1.05: 1 molar composition in an ice bath to control the heat of the exothermic acid-base reaction. The neat amine was added dropwise very slowly (roughly 0.5 mL/h) by a pressure-equalizing dropping funnel (10 mL) to the neat acid (HTFSA) in a two-neck round bottom flask (100 mL) and mixed vigorously via magnetic stirrer (3000 rpm) to suppress the evolution of the localized heat. During mixing, care must be taken as primary amine-PILs tend to solidify at low temperature; therefore, to ensure homogeneous mixing, the reaction vessel was occasionally taken out of the coolant while stopping the addition of the amine to the mixture and then mixed at room temperature by magnetic stirrer for several minutes. After that, the reaction vessel was put back in the coolant and addition of amine was restarted with the same rate as previous. In this way, colourless PILs was achieved efficiently.

Also, in each case, around 15.0 g/batch of PILs was synthesized. To increase the batch size, a 2nd batch of 15.0 g PILs could be prepared in the same reaction vessel simply by adding acid in the already prepared PILs from the 1st batch with the subsequent addition of amine per the previous procedure.

In our observation, if a large amount of HTFSA/$[TFSA]^-$ is present in the flask (100 mL), then it was very difficult to control the residual heat and thus instead of transparent PILs, coloured sample was obtained. So, we recommend that during synthesis depending on the targeted batch size, reaction apparatus and synthetic condition, i.e., rate of addition should be carefully optimized as the purity of the product is very sensitive to the heat evolved during the reaction.

In general, after the preparation of $[TFSA]^-$ based PILs, the excess amine was removed by vacuum drying at 80 ^oC (24 h). But, for [2-Mpra][TFSA], [N-E*i*pra][TFSA] and [Dema][TFSA], the highest temperature for drying was 60 ^oC and was constant for 24 h. In all cases, the temperature for drying was gradually raised from 30 ^oC (6h) to the highest temperature.

In the case of other primary alkylamine-PILs based on oxo acids, both neat amine and acid was added simultaneously via pressure-equalizing dropping funnel in a three-neck round bottom flask. In such case, solid product was obtained so to mix them properly, small amount of acetonitrile was added as solvent and the mixture was mixed again overnight in a room temperature water bath. After that the solvent was removed by vacuum drying at 50 ^oC for 24 h. Due to the high melting point of these class of PILs, final temperature of drying was set at 100 ^oC for 48 h.

Finally, the PILs were kept in an Ar atmosphere glove box (VAC, $[O_2]$ < 1 ppm, $[H_2O]$ < 1 ppm). The water contents of PILs were determined to be below 200 ppm by Karl-Fischer titration.

Physicochemical characterization of protic ionic liquids

Phase analysis were done via a DSC 7020 differential scanning calorimeter (Hitachi, Japan) under an N_2 atmosphere. In an Ar atmosphere inside of a dry glove box, the samples were sealed in aluminium pans. Then, the samples were subjected to the following thermo-profile in which temperature was scanned at first from 150 ^{o}C to -150 ^{o}C, then again from -150 ^{o}C to 150 ^{o}C (rate of heating/cooling: 10 ^{o}C min^{-1}). DSC thermogram was recorded while performing the reheating scans.

Thermogravimetric measurements were conducted using a TG-DTA 7200 thermo-gravimetry/differential thermal analyzer (Hitachi, Japan) from room temperature to 550 ^{o}C at a heating rate of 10 ^{o}C min^{-1} under an N_2 atmosphere with open aluminium pans. The decomposition temperature (T_d) was determined as the temperature at which 5% mass loss was began.

FT-IR spectra were recorded on a Nicolet iS50 FT-IR spectrometer (Thermo-scientific, USA) in the 1000–4000 cm^{-1} range using CaF_2 disks at room temperature. Samples for IR spectra were prepared inside the dry box.

The viscosity was measured via a Physica MCR 301 rheometer (Anton Paar, Austria) using a CP501 cone plate (50 mm in diameter, 1° angle) under dry air conditions at a temperature controlled in the range of 25 ^{o}C–100 ^{o}C. At zero shear rate, the sample was equilibrated at each temperature for 15 minutes prior to the measurement. Then, a steady preshear was applied at a shear rate of 1 s^{-1} for 60 s followed by a 120 s rest period before each measurement to remove any previous shear histories. The viscosity value was taken at zero shear rate. For, shear thinning behaviour, shear rate was varied from 10 s^{-1} to 8000 s^{-1}. At temperatures above 25 ^{o}C, viscosity of the sample reduces greatly which in turn results the expulsion of samples under very high shear rate, i.e., 8000 s^{-1}. Thus, the highest shear rate was kept at 4000 s^{-1} for measurements at 40 ^{o}C, 60 ^{o}C, 80 ^{o}C, and 100 ^{o}C.

Conductivities were obtained by deploying a CG-511 B electrical conductivity cell (DKK-TOA corporation, Japan). The cell was comprised of two platinum black electrodes. Prior to the measurement, the cell was cleaned by water and ethanol respectively for three times each. After drying, the conductivity cell was immersed in a test tube containing ~1.5 mL of PILs. Then, the complex impedance spectra were measured using a Biologic VMP2 multi-potentiostat (Biologic, France) in the frequency range of 1 Hz to 1 MHz. The cell constant of the conductivity cell was determined with a solution of 0.01 M KCl at 25 ^{o}C. And, the impedance spectra of respective PILs were measured from 30 ^{o}C to 100 ^{o}C using a ESPEC SU-261 constant temperature oven (ESPEC, Japan). Prior to the measurement of impedance spectra, the samples were thermally equilibrated at each temperature for ~1 h. After acquiring the spectra, the conductance was determined from the actual axis touchdown point in the Nyquist plots of impedance data.

A DA-100 thermoregulated density/specific gravity meter (Kyoto Electronics Manufacturing Co. Ltd., Japan) was used to measure the density in the range of 15 ^{o}C–40 ^{o}C. For high melting temperature PILs, the measurement was hindered by the limitation of the instrument.

^{1}H-NMR spectra were acquired with a JEOL JNM-AL 400 NMR spectrometer (JEOL, Japan) using a SC-002 5-mm coaxial capillary (Shigemi, Tokyo) with the sample in the inner tube and the reference solvent (DMSO-d_6 having 1% TMS) in the outer tube. ^{1}H chemical shifts were externally referenced to the TMS peak. The NMR data was processed by using a data analysis software ALICE 2, version 6 (JEOL, Japan).

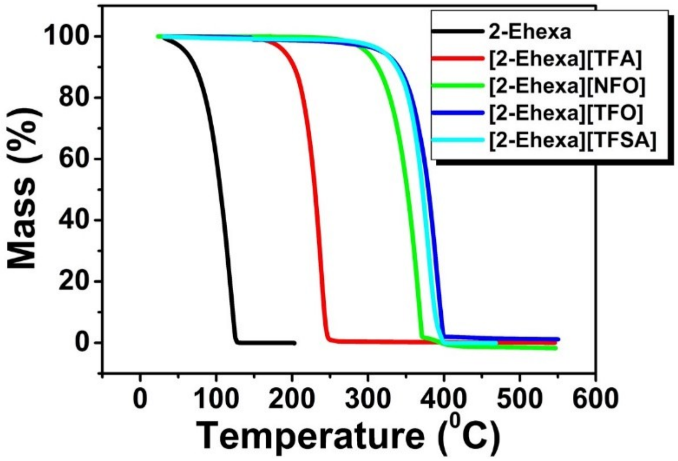

Fig. S1. Thermogravimetry curves of primary alkylamine-PILs showing the effect of anionic structure.

PILs/PSs	T_d (°C)	T_m (°C)
**[2-Mbua][TFSA]**	305.0	20.7
**[N-E*i*pra [TFSA]**	315.6	13.6
**[Dema][TFSA]**	325.1	25.0
**[2-Ehexa][TFSA]**	319.6	-1.4
**[2-Mpra][TFSA]**	300.2	59.4
**[2-Ehexa][TFO]**	318.7	138.6
**[2-Ehexa][NFO]**	297.6	168.2
**[2-Ehexa][TFA]**	189.6	-36.1 (T_g)

Table S1. Thermal properties of all the PILs/PSs synthesized in this work.

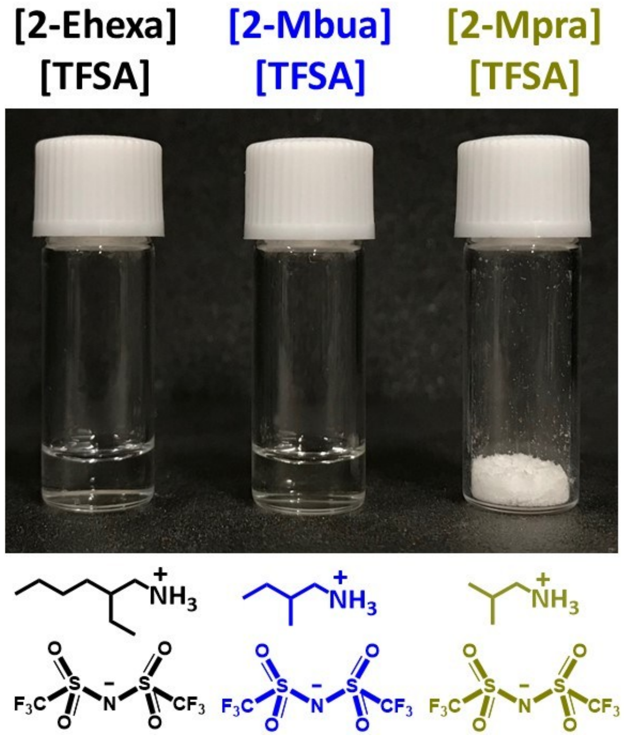

Fig. S2. Primary alkylamine-PILs with the variation in the alkyl chain length of cations.

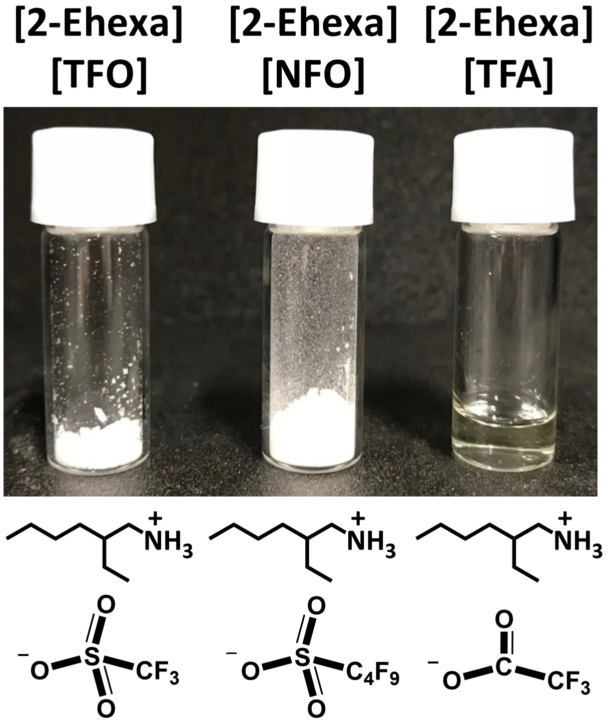

Fig. S3. 2-Ethylhexylamine based PILs/PSs with different oxo-acids.

Aside from the $[TFSA]^-$ salt, further investigation of 2-Ehexa based PILs resulted in only one liquid product, that is, [2-Ehexa][TFA] due to reduction in ΔpK_a as both [2-Ehexa][TFO] and [2-Ehexa][NFO] were high melting PSs (**Fig. S3**). With the decrease of ΔpK_a, in primary alkylamine-PILs, strength of N-H bond became weaker thereby reducing the ion-pair interaction energy as observed for tertiary alkylamine-PILs.[1] Therefore, change in phase behaviour was noticed.

Although not included in this work, we attempted several other branched alkylamines in combination with $[TFO]^-$ which did not yield room temperature liquids. Therefore, although true reason for such contrasting phase behaviour is still unknown, H-bonds have the most significant effect in the phase transition of primary alkylamine-PILs as observed in EAN analogues.[2-4]

At high ΔpK_a, conformational robustness[5] of $[TFSA]^-$ than $[TFO]^-$ was crucial to off-set the interaction energy and in turn efficient ion-packing.

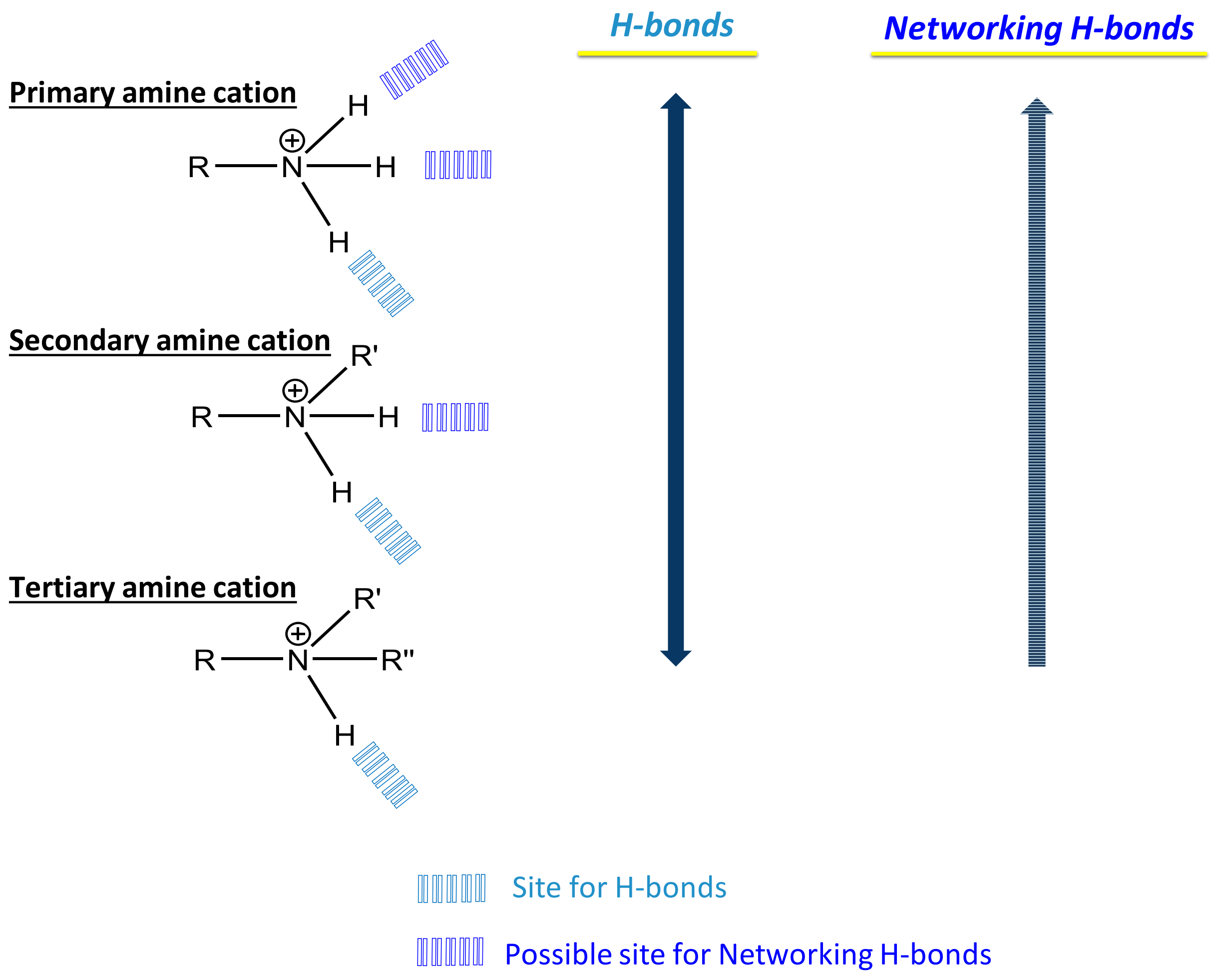

Fig. S4. Possible interaction sites for H-bonds and networking H-bonds in protic ionic liquids depending on the number of H-bond donors in the structure of cation.

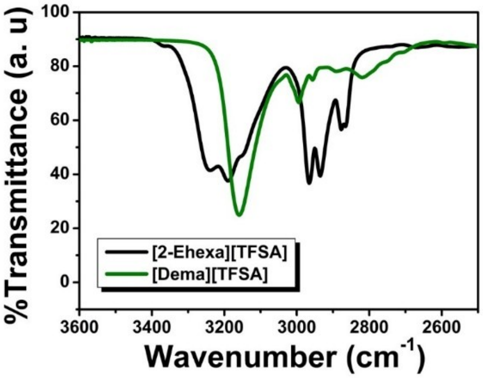

Fig. S5. FT-IR spectra of [2-Ehexa][TFSA] in comparison with [Dema][TFSA] at room temperature.

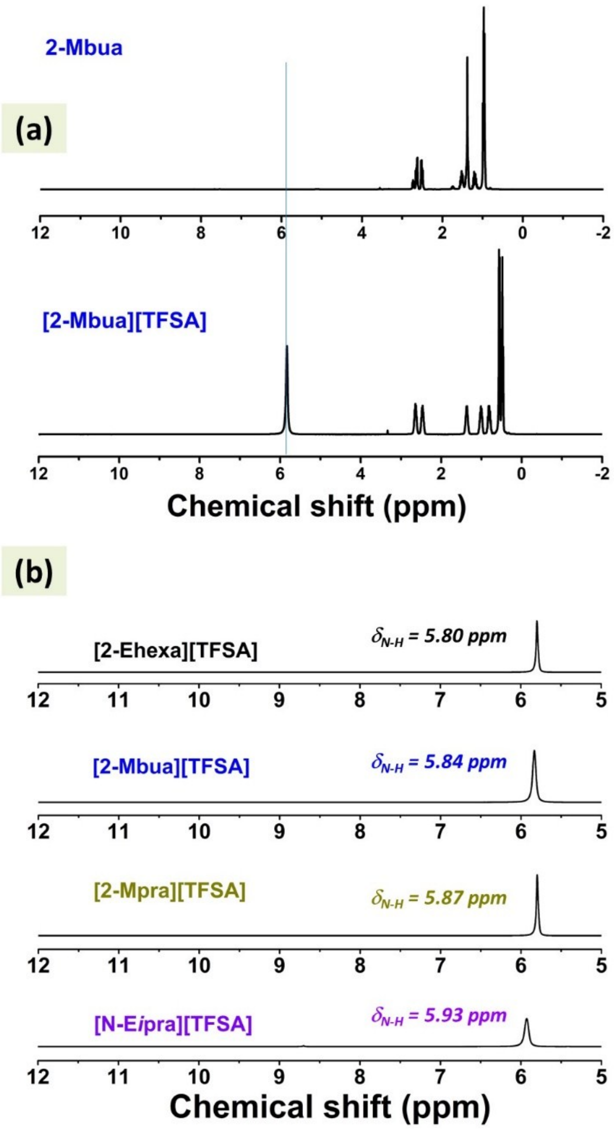

Fig. S6. ^{1}H-NMR spectra of (a) primary amine (neutral) and the corresponding PILs, (b) PILs with primary and secondary amine cations measured at 40 ^{o}C.

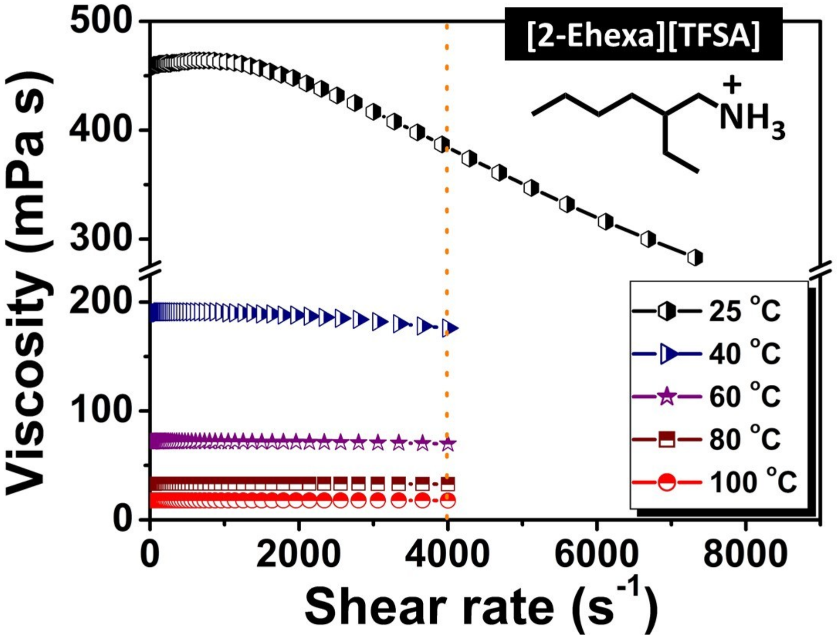

Fig. S7. Change of viscosity as a function of shear rate under various temperatures. Shear rate was limited to 4000 s^{-1} from 40 °C due to the expelling of the samples at very high shear rate.

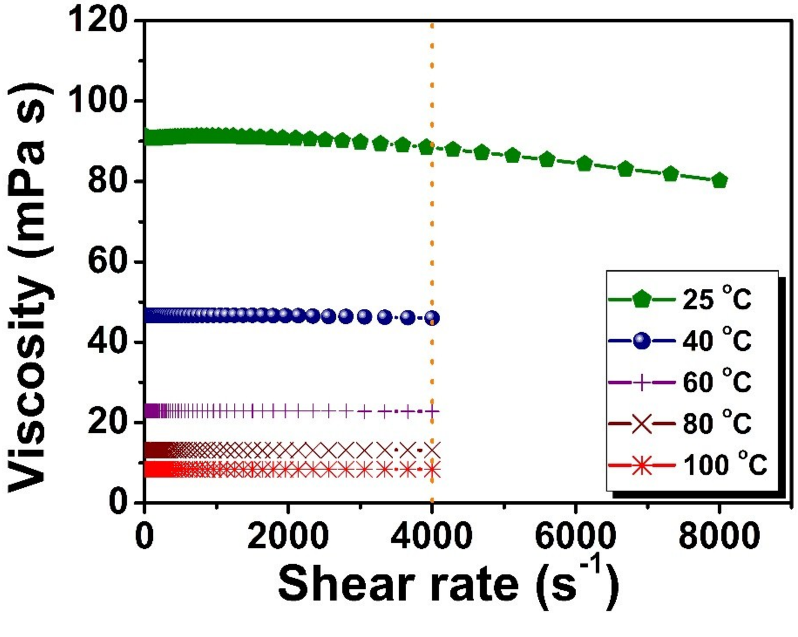

Fig. S8. Change of viscosity as a function of shear rate under various temperatures. Shear rate was limited to 4000 s^{-1} from 40 °C due to the expelling of the samples at very high shear rate.

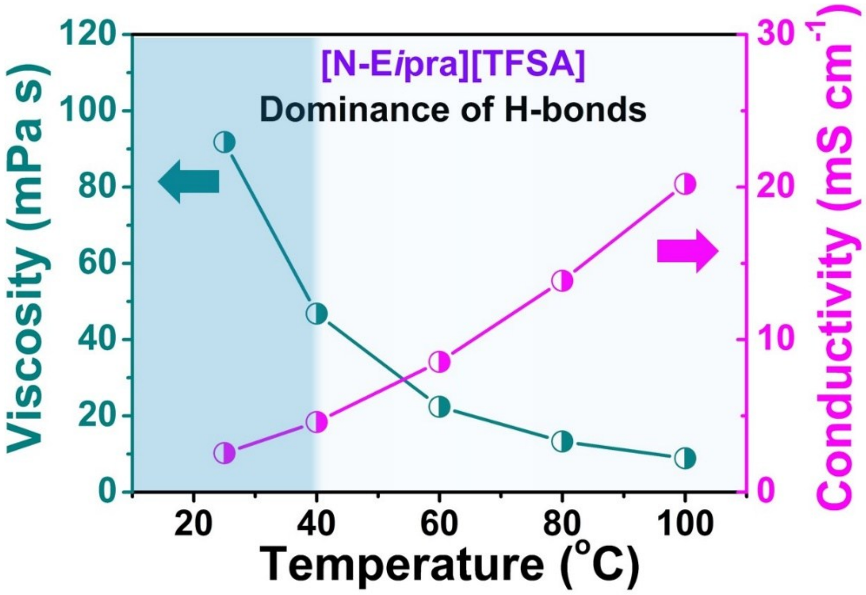

Fig. S9. Change of viscosity and conductivity as a function of temperature.

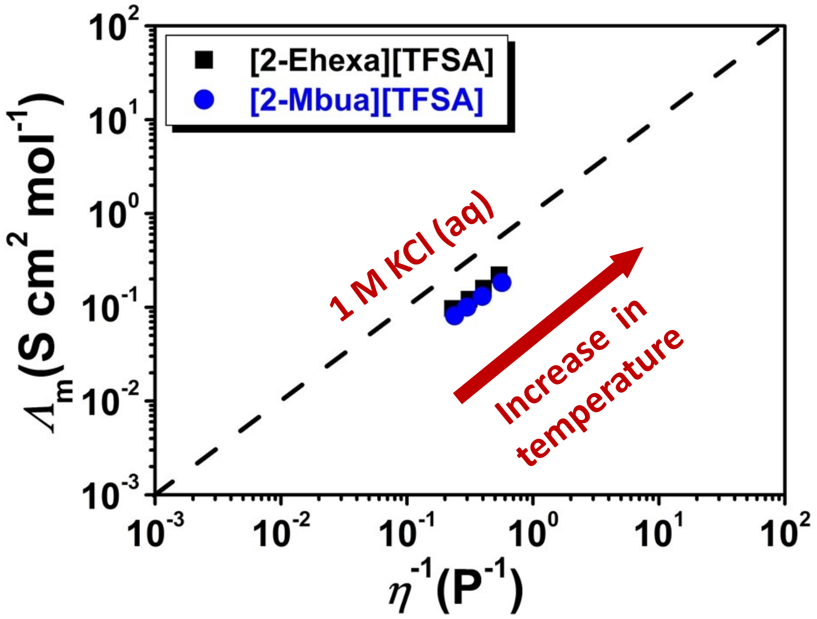

Fig. S10. Walden plot (25 °C–40 °C).

<u>**References**</u>

1. K. Fumino, V. Fossog, K. Wittler, R. Hempelmann, and R.Ludwig. *Angew. Chem. Int. Ed.*, 2013, **52**, 2368–2372.
2. R. Hayes, S. Imberti, G. G. Warr, and R. Atkin, *Angew. Chem. Int. Ed.*, 2013, **52**, 4623–4627.
3. A. Mondal, and S. Balasubramanian, *J. Phys. Chem. B*, 2015, **119**, 1994–2002.

4. R. Hayes, G. G. Warr, and R. Atkin, *Chem. Rev.*, 2015, **115**, 6357−6426.
5. J. N. C. Lopes, K. Shimizu, A. A. H. Pa´dua, Y. Umebayashi, S. Fukuda, K. Fujii, and S.-I. Ishiguro, *J. Phys. Chem. B*, 2008, **112**, 1465-1472.
